# The Effects of Growth Modification on Pollen Development in Spring Barley (*Hordeum vulgare* L.) Genotypes with Contrasting Drought Tolerance

**DOI:** 10.3390/cells12121656

**Published:** 2023-06-18

**Authors:** Piotr Ogrodowicz, Maria Katarzyna Wojciechowicz, Anetta Kuczyńska, Paweł Krajewski, Michał Kempa

**Affiliations:** 1 Institute of Plant Genetics Polish Academy of Sciences, 34 Strzeszynska Street, 60-479 Poznan, Poland; akuc@igr.poznan.pl (A.K.); pkra@igr.poznan.pl (P.K.); mkem@igr.poznan.pl (M.K.); 2 Department of General Botany, Institute of Experimental Biology, Faculty of Biology, Adam Mickiewicz University, 1 Wieniawskiego Street, 60-479 Poznan, Poland; maria.wojciechowicz@amu.edu.pl

**Keywords:** drought, growth modifications, transcription factor, light deprivation, pollen development

## Abstract

Drought stress inducing pollen sterility can reduce crop yield worldwide. The regulatory crosstalk associated with the effects of drought on pollen formation at the cellular level has not been explored in detail so far. In this study, we performed morphological and cytoembryological analysis of anther perturbations and examined pollen development in two spring barley genotypes that differ in earliness and drought tolerance. The Syrian breeding line CamB (drought-tolerant) and the European cultivar Lubuski (drought-sensitive) were used as experimental materials to analyze the drought-induced changes in yield performance, chlorophyll fluorescence kinetics, the pollen grain micromorphology and ultrastructure during critical stages of plant development. In addition, fluctuations in *HvGAMYB* expression were studied, as this transcription factor is closely associated with the development of the anther. In the experiments, the studied plants were affected by drought, as was confirmed by the analyses of yield performance and chlorophyll fluorescence kinetics. However, contrary to our expectations, the pollen development of plants grown under specific conditions was not severely affected. The results also suggest that growth modification, as well as the perturbation in light distribution, can affect the *HvGAMYB* expression. This study demonstrated that the duration of the vegetation period can influence plant drought responses and, as a consequence, the processes associated with pollen development as every growth modification changes the dynamics of drought effects as well as the duration of plant exposition to drought.

## 1. Introduction

Pollen development is an important process in the life cycle of plants, because only appropriately formed pollen guarantees the proper formation of full-sized seeds. However, under unfavorable conditions, the anther development is disturbed, which leads to the formation of sterile pollen [1]. Elevated temperatures and frequent episodes of drought driven by climate change affect developmental and physiological processes in plants, and ultimately crop yield and quality [2]. Drought stress also inhibits the growth of plants by influencing their various biochemical functions such as photosynthesis, chlorophyll synthesis, nutrient metabolism, ion uptake and translocation, respiration, and carbohydrate metabolism [3,4]. Moreover, it affects pollen development by disturbing the internal homeostasis of plant cells, possibly by altering the intracellular levels of sugars, hormones, and reactive oxygen species [5,6]. Drought stress and elevated temperatures, as detrimental abiotic factors that limit crop productivity, ultimately have a negative impact on food security worldwide [4] and exacerbate the effects of rapid climate change on agricultural production.

However, the extent of damages, the recovery capacity, and the impact on the yield depend on the developmental stage at which the crop is exposed to this abiotic stress [7]. Although the impact of stress on plant yield and development has been extensively studied [8,9], the interactions of stress responses with plant phenology have often been neglected.

Pollen development in the anthers has been well studied [10,11]. Because of its role in anther and pollen development, the growth regulator gibberellic acid (GA) has been studied in several papers. GA is essential for anther development and pollen viability [12], and its signal transduction occurs predominantly in the tapetal cells [13]. *GAMYB* appears to be the major transcription factor in the GA signaling pathway [14,15,16]. The interactions between gibberellin and photoperiod signaling pathways and the potential gene integrators have been studied in various plant species [17,18,19]. Although studies have analyzed various *MYB* genes based on their involvement in regulating the response to abiotic stress [20,21], the role of *HvGAMYB* in drought response in the context of plant phenology remains unclear.

To survive in drought conditions, plants develop a few strategies to cope with limited water availability. These strategies include (i) drought avoidance [22], (ii) drought tolerance [23], (iii) drought resistance [24], (iv) drought abandonment [25], and (v) drought-prone activation of biochemical/physiological traits [26]. In addition to these, (vi) drought escape (DE) is an important strategy adopted by plants to cope with impending unfavorable environmental conditions. However, classifying plants based on their drought response mechanism is not entirely accurate since the timing of the onset of the mechanism varies from one plant to another, such as before or after the occurrence of a drought event. This implies that plants use a combination of DE and other strategies to survive (and reproduce) under drought. Although DE is considered a characteristic of “ephemeral native plants”, major cereals such as wheat and barley may exhibit a mechanism similar to DE, which is referred to as “earliness” or “early flowering” [27].

Both light and darkness act as environmental stimuli regulating plant growth and development from seedling emergence to senescence. Darkness induces the secretion of phytohormones such as GA and ethylene in germinating seeds [28]. Under darkness, GA accumulates and binds to its receptor gibberellin-insensitive dwarf 1 as well as its targets, DELLAs (GAI and RGA), which are the negative regulators of GA signal transduction [29]. Detailed studies on gene expression patterns have revealed various discrepancies between dark-induced and developmentally controlled processes [30,31,32]. 

As increasing evidence suggests that stress responses depend on the developmental stage of the plant [33], we decided to study specific developmental stages to understand the extent of the negative influence of stress on plant development. Because barley (*Hordeum vulgare* L.) is usually used as a model plant in studies on abiotic stress responses of plants [34] and precision developmental scales are currently available for barley [35], we designed the experiment with two spring barley genotypes that differ in phenology and resistance to abiotic stresses [36,37]. Experiments were designed from a multi-perspective approach to enrich the knowledge about better adaptations of plants to stressful growth conditions.

Our study aimed to (i) study the effects of drought on spikes fertility, (ii) explore drought-induced alterations in pollen grain ultrastructure, and (iii) characterize the expression pattern of *HvGAMYB* under light deprivation and drought conditions in phenologically differentiated barley plants.

## 2. Materials and Methods

### 2.1. Plant Material

Two barley genotypes were used in this investigation: Lubuski is an old Polish cultivar derived from a Heines-Haisa/Skrzeszowicki hybrid, and Cam/B1/CI08887//CI05761 (hereafter referred to as CamB) is a Syrian breeding line provided to Dr. A. Górny (IPG PAS) by Drs. S. Grando and S. Ceccarelli of ICARDA in Aleppo. The two cultivars were selected because they have different phenology and are adapted to different environments (Lubuski, a late-heading cultivar grown in Poland; CamB, an early-heading line adapted to dry conditions) [36,38].

### 2.2. Growth Conditions and Experimental Designs

The growth conditions were described in detail in [37]. Five seeds from each of the accessions were sown in plastic pots (40 cm × 26 cm × 26 cm) filled with arable soil and peat (3:1, *w*/*w*), and the plants were cultivated under optimal conditions: temperature of 22 °C/18 °C day/night, humidity of 50–60%, and photoperiod of 16/8 h light/dark. Each treatment was conducted in triplicate. All experiments were performed in growth chambers under fully controlled conditions (IPG PAS phytotrons).

In this study, the barley external development stages scale [35], in which the later stages from the Zadoks scale [39]—decimal code, which defines specific developmental stages that are easy to identify in the field (Supplementary Table S1)—have been replaced by the last flag elongation (LFE) stages to provide a clear system for developmental staging associated with reproductive development. Samples (anthers and pollen grains) were collected at LFE1 (flag leaf emerged completely and unrolling, ligule may be visible; last flag sheath extended 0.5–5 cm—development point 1), at LFE3 (opening of the flag leaf and awns are visible; the last flag sheath is more than 10 cm long; the rachis begins to elongate and move up the spike toward the last flag sheath) and at LFE4 (spike has completed its upward movement and was entirely localized within the last flag sheath—development point 2) stages. 

### 2.3. Drought Treatment

Plants were irrigated until flag leaves appeared (Z37) and then subjected to two irrigation treatments: (i) control condition (soil was maintained at ~70% of field capacity (FC)); and (ii) drought stress (at 20% FC) following a previously published procedure [40]. Soil moisture was monitored by gravimetric method and—additionally—evaluated volumetrically (if necessary) with the FOM/mts device [36,41].

### 2.4. GA3 and TRinexapac Application

The application protocols of growth regulators were described in [37]. In general, the modified methods implemented by [42,43] were used in this investigation for GA3 and Trinexapac-ethyl applications, respectively. The bioactive GA3 solution (Sigma-Aldrich, Burlington, MA, USA) was prepared by dissolving the powder in distilled water. For each plant, 1 mL of GA3 solution was used. Trinexapac-ethyl (TR), a growth regulator that belongs to the cyclohexanedione group was used as the commercial product Moddus 250 EC (Syngenta, Wilmington, DE, USA). Its mode of action involves the suppression of gibberellin biosynthesis. Both GA3 and TR solutions were sprayed directly onto the leaves at the beginning of tillering stage (Z21—Zadoks Stage). For the control plants, distilled water was sprayed. 

### 2.5. Light/Dark Cycle Treatment

In this study four types of treatments were applied: C—control condition; D—drought condition; D+GA—drought condition combined with GA3 application; D+TR—drought condition combined with TRinexapac application, in two experiments—experiment 1 (hereafter referred to as Exp 1), and experiment 2 (hereafter referred to as Exp 2). In Exp 2, an additional stress application—light deprivation was implemented. The dark conditions (started at the beginning of abiotic stress—Z37) were achieved by carefully covering the barley plants in a dark growth chamber. The dark treatment was carried out for 7 days and covered the beginning of the flowering period. After the dark treatment, the plants were exposed to the normal light regime.

### 2.6. Phenology and Phenotypic Evaluation

In this study, four developmental stages (tillering, flag leaf, flowering, heading) appearances were observed when for at least 51% of all plants grown under given experimental variants the developmental phase was observed and presented as a number of days after sowing (DAS). 14 yield-related traits were also investigated. Name of these traits with abbreviations and descriptions of measurement methods are given in Table 1. 

### 2.7. Anther Morphology Evaluation

Anther measurements were performed using a stereomicroscope (Motic SMZ-161) following a previously described protocol [44]. Images of the anthers were captured at fixed magnification using a digital camera (Moticam CMOS BTU8). Images were measured using Motic Advanced 3.2 software (Xiamen, China). Anther length (mm) was determined after the removal of anthers from the primary flower of the largest spikelet. Anther width (mm) was the width of the anthers at their widest point. Samples were taken at time LFE3, just before flowering. 

### 2.8. Pollen Viability and Morphology Evaluation

Pollen viability and fertility were evaluated using KI/I2 (method 1—abbreviated as Pv1) and TTC (2,3,5-Triphenyl Tetrazolium Chloride) (method 2—abbreviated as Pv2) staining methods as described in [45] and in [46], respectively, with minor modifications. Only mature pollen was used for the evaluations (LFE4). Anthers from different plants were used for each replicate. Pollen was extracted following the previously published protocol [47]. The detailed protocols of pollen viability evaluations implemented in this study were presented in [37]. 

The pollen grain area, perimeter, width (the widest point) and length were evaluated. The morphological evaluations were based on 100 randomly selected pollen grains observed in each genotype in each treatment.

### 2.9. Chlorophyll Fluorescence Measurements 

Chlorophyll content and fluorescence were measured on both control and stress plants. Data were collected at two development points—LFE1 and LFE3—always at the same time of day (09:00). Measurements were performed using a FluorPen FP 100-MAX (Photon Systems Instruments, Drasov, Czech Republic). Fluorescence transients for chlorophyll-a were recorded in the center of the completely spent leaf (second leaf from the top). Leaves were allowed to adjust to the darkness for 30 min before measurements were made using leaf clamps provided by the manufacturer. Leaves were then exposed to a pulse of saturating light at an intensity of 3000 μmol m^−2^ s^−1^, and all studied parameters were analyzed. Nine replicates were analyzed for each cultivar and treatment (three leaves from three plants/treatment). The parameters analyzed in this study are listed in Table 2.

### 2.10. Relative Water Content (RWC)

The RWC was calculated according to the method of Barrs and Weatherley [48] using the formula RWC (%) = (FW − DW)/(TW − DW) × 100, where: FW is the fresh weight of the detached second leaf; TW is the turgid weight of the second leaf, which was incubated in distilled water for 24 h in darkness after detachment; and DW is the dry weight of the second leaf after it was dried in a dryer at 60 °C for 48 h. Nine replicates (three replicates from three plants/treatments) were determined for each combination of genotype and treatment. The samples were collected at LFE3.

### 2.11. Microscopic Observations

For cytological studies, anthers from spikelets of the middle zone of the spike (three per each experimental treatment) were collected. Isolated anthers were fixed in a mixture of equal volume of 2.0% glutaraldehyde and 2.0% paraformaldehyde in 0.05 M cacodylic buffer. The dates of sample collection were shown in Supplementary Table S2. 

### 2.12. Light Microscopy Observation

The anthers were collected at LFE4. For observations in a light microscope (LM) commercially available acrylic resin Technovit 7100 (Heraeus Kulzer Gmbh, Wahrheim, Germany) was used. Fixed samples were dehydrated in ethanol series (from 5% to 100%) and embedded in Technovit resin. Sections 4 µm thick were stained with 0.05 toluidine blue in 1% sodium borate. Micrographs were taken using a light microscope (Axioscope A1, Zeiss, Germany) with an attached AxioCam MRc5 digital camera (Carl Zeiss GmbH, Jena, Germany). On average, eight anthers from each experimental treatment were examined. 

### 2.13. Electron Microscopy Observation 

The probes were collected at LFE4. For observations in the transmission electron microscope (TEM) the fixed anthers were rinsed three times in 0.05 M cacodylic buffer (pH 6.8), post-fixed for two hours in 1% osmium tetroxide at room temperature, and counter-stained with 2% aqueous uranyl acetate (pH 5.0). Samples were dehydrated in a graded acetone series (from 5% to 100%) and embedded in low-viscosity Spurr’s resin (Polysciences). The sections of 90 nm in thickness were counter-stained for twenty minutes in lead citrate and observed with JEOL JEM 1200 EXII (Jeol, Tokyo, Japan) transmission electron microscope at an acceleration of 80 kV. At least five anthers from each experimental treatment were examined. Moreover, from the same samples, semithin sections were collected and observed microscopically (Axioscope A1, Zeiss, Germany) and documented with an attached AxioCam MRc5 digital camera (Carl Zeiss GmbH, Jena, Germany). 

### 2.14. RNA Extraction, Reverse Transcription, and Real-Time PCR

Anther tissue was collected at two developmental stages (LFE1 and LFE4). For each stage, four biological replicates were collected, comprising approx. 100 anthers from four individual spikes. The RNA extraction procedure and RT-qPCR protocol were described in [37]. Briefly, the RNA was extracted using the RNeasy Mini Kit (QIAGEN, Hilden, Germany) according to the manufacturer’s protocol (QIAGEN, Germany). All isolated RNA samples were treated with TURBO DNase (Thermo Fisher Scientific, Waltham, MA, USA) according to the manufacturer’s instructions to exclude trace contamination of samples with genomic DNA. The purity of all RNA samples was assessed via OD260/280 and OD260/230 absorbance ratios and their structural integrity was evaluated using denaturing agarose gel electrophoresis. All RNA samples were adjusted to the same concentration (100 ng/μL). Single-stranded cDNA was synthesized from 1 μg of total RNA using the iTaq Universal SYBR Green One-Step Kit according to the manufacturer’s instructions. RT-qPCR was performed using the CFX Connect Real-time PCR Detection System (Bio-Rad). The real-time quantitative PCR analysis performed in this study met MIQE criteria [49]. Data were normalized using three stable reference genes: *UBI* (GenBank ID: M60175.1), *ACT1* (GenBank ID: AY145451.1), *UPL* (GenBank ID: XM_045123725.1). Relative changes in gene expression were calculated using the comparative 2^−ΔΔCt^ method and normalized to the corresponding reference genes [50]. *HvGAMYB* primers were designed by using Primer3 (https://primer3.org/) (accessed on 19 April 2023). The list of primers and probes used in this study is shown in Supplementary Table S3.

### 2.15. Statistical Analysis

Analysis of variance (ANOVA) for observed quantitative traits was performed in a model containing fixed effects of genotypes (CamB, Lubuski—G), treatment (T), development point (DP) (for chlorophyll fluorescence parameters), G × T interaction, G × DP interaction (for chlorophyll fluorescence parameters) and T × DP interaction (for chlorophyll fluorescence parameters). Significant sources of variation in ANOVA were selected at *p* < 0.001 (approximate threshold resulting from the application of the Bonferroni correction concerning multiple testing for all traits). Multiple comparisons of mean values were made using Fisher’s Protected Least Significant Difference (FPLSD) test at *p* < 0.05. In the case of physiological parameters, due to non-normal distributions of observed variables, analysis of variance was performed on the data transformed by optimal Box-Cox transformation [51]. Statistical computations and visualizations were performed in Genstat 22 [52].

## 3. Results

### 3.1. Differences in Plant Phenology, Chlorophyll Fluorescence Kinetics and Yield Reduction in Drought

#### 3.1.1. Phenology

**Figure 1:**
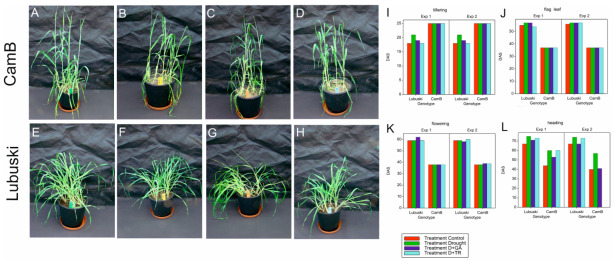
Phenological differentiation between the studied genotypes. Photos showing plants sowed at the same time (0 DAS) and subjected to different water regimes: CamB plants grown under C condition (**A**), D condition (**B**), D+GA condition (**C**), and D+TR condition (**D**) and Lubuski plants grown under C condition (**E**), D condition (**F**), D+GA condition (**G**), and D+TR condition (**H**). The development pattern of the studied genotypes representing different times (DAS) of tillering (**I**), flag leaf (**J**), flowering (**K**), and heading (**L**) stages. DAS—days after sowing.

The results of the analysis of variance (ANOVA) showed significant differences (at *p* < 0.001) between the studied genotypes for the time of reaching two phenological stages —flag leaf stage (Z38) and flowering (Z65)—in both types of experiments (Supplementary Table S4). Tillering process was initiated much earlier in the late-heading Lubuski genotype, whereas both flag leaf stage and flowering were reached earlier in CamB than in Lubuski. In general, the applied treatments caused the delay of plant development with two exceptions (a similar number of DAS was recorded in the heading for both CamB and Lubuski plants subjected to C and D+GA conditions in Exp 2) (Figure 1). 

#### 3.1.2. Yield Performance

The results of ANOVA revealed significant effects of the applied treatments on almost all the investigated yield-related traits (except for NSSm and NSSl) in Exp 1 (Supplementary Table S4). In Exp 2, significant effects of treatment were observed only for four investigated traits (Tn, WGSm, NSSl, TGW). In Exp 1, significant genotypic effects were recorded for traits linked to the main spike (LSm, NSSm, NGSm, WGSm) and lateral spike (LSl, NSSl) morphology as well as for PTn (Supplementary Table S3). In the second experiment, the studied genotypes showed significant differences in all the same yield-related traits with one exception (PTn). In addition, in Exp 2, a significant effect of genotype was also noticed for FSl. The mean values of the nine studied traits recorded in both types of experiments are presented in Supplementary Figure S1. The means of most of the studied traits were significantly reduced under drought conditions in Exp 1 (except for Tn and PTn). Greater mean values of PTn were recorded for CamB in the D condition (Exp 1) compared to the mean values of this trait recorded for Lubuski. In Exp 2, greater mean values (compared to the C condition) of traits associated with the morphology of the main spike (NSSm, NGSm, WGSm) as well as lateral spike (NSSl, NGSl, WGSl) were recorded in the D condition for CamB.

#### 3.1.3. Chlorophyll Fluorescence Kinetics and RWC

The results of ANOVA from Exp 1 showed significant effects of genotype for ABS_RC, TRo_RC, ETo_RC, Dlo_RC, and Pi_Abs. Significant effects of DP on all chlorophyll fluorescence parameters were noted in both Exp 1 (except for ETo_RC) and Exp 2 (Supplementary Table S4). In Exp 2, significant effects of the applied treatment were observed for almost all the studied traits (except for Φ_Eo). Significant effects of G × T interaction were recorded for ETo_RC, Ψ_o, and Φ_Eo in Exp 1 and for ABS_RC, TRo_RC, Dlo_RC, Φ_Do, and Pi_Abs in Exp 2. The mean values recorded for chlorophyll fluorescence parameters in both types of experiments are presented in Supplementary Figure S2. The results of ANOVA from both Exp 1 and Exp 2 showed no significant effects of genotypes on relative water content (RWC); this is contrary to the highly significant effects of the applied treatments on this trait observed in both types of experiments (Supplementary Table S4). For both the genotypes, higher mean RWC values were observed in the C condition compared to the values recorded in stress conditions in Exp 1 and Exp 2 (Supplementary Figure S3). In Exp 2, higher mean RWC values were observed for CamB in the D condition compared to the values recorded for Lubuski in this condition. 

### 3.2. Spike Fertility and Anther Morphology

The results of ANOVA from Exp 1 showed significant effects of the treatments on FSm and FSl and the significant effect of G × T interaction on FSm (Supplementary Table S4). In Exp 2, significant effects of genotype and G × T interaction on FSl were recorded. No significant effects of treatments were observed in this experiment for spike fertility-related traits.

**Figure 2:**
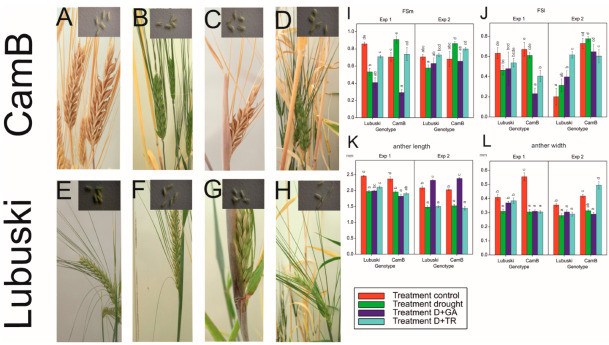
Photos showing the differences in the morphology of spikes and anthers among the studied plants subjected to different water regimes in Exp 1: spikes and anthers of CamB plants grown under C condition (**A**), D condition (**B**), D+GA condition (**C**), and D+TR condition (**D**) and spikes and anthers of Lubuski plants grown under C condition (**E**), D condition (**F**), D+GA condition (**G**), and D+TR condition (**H**). Mean values (with standard errors) of FSm (**I**), FSl (**J**), anther length (**K**), and width (**L**) recorded for the studied genotypes under four applied treatments. Letters denote groups of similar mean values obtained by the FPLSD test at *p* < 0.05.

Lubuski showed higher mean FSm values in the C condition compared to the Syrian genotype in Exp 1, but no significant differences between the studied genotypes were noticed for this trait in the C condition in Exp 2 (Figure 2). In the D condition, a rapid increase of FSm values was observed in CamB in both types of experiments. The application of growth regulator (GA) in the D condition resulted in decreases in the mean values of FSm compared to the condition where drought was applied alone for both studied genotypes (but no significant differences in mean FSm values between the D and D+GA condition were recorded for Lubuski in Exp 2). For Lubuski, lower mean FSm values were recorded in the C condition in Exp 1 compared to the C condition in Exp 2. For CamB, higher mean values of FSl were observed under the D conditions in both experiments compared to the late-heading genotype. For the Syrian genotype, higher mean FSl values were noticed under all types of treatments (except for D+TR) in Exp 2 compared to Lubuski. 

The ANOVA results revealed the significant effects of treatment on anther length and width. Significant effects of G × T interaction were recorded only for anther width—in the C condition, a much higher anther width was observed for CamB (Figure 2L). 

### 3.3. Pollen Micromorphology and Ultrastructure

#### 3.3.1. Pollen Grain Morphology and Viability

**Figure 3:**
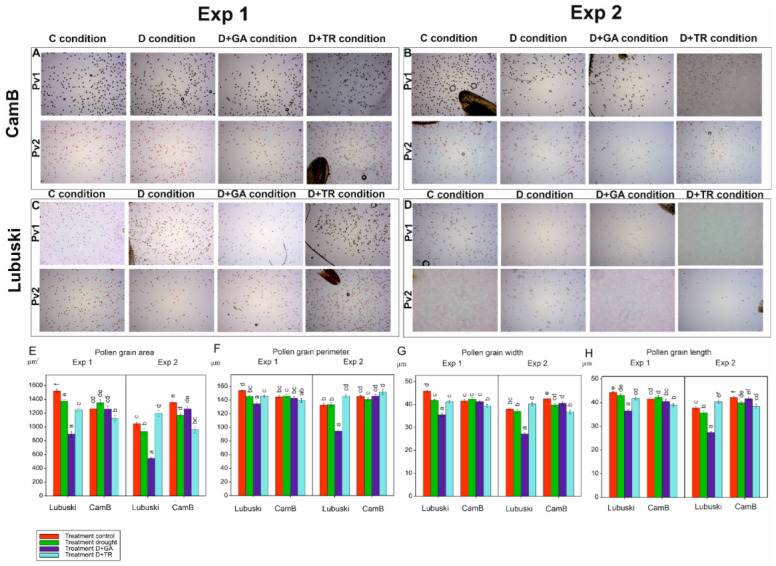
Images presenting the LM analysis results obtained using two different methods of pollen viability evaluation (Pv1 and Pv2): pollen grains collected from CamB plants grown under four types of water regime in Exp 1 (**A**) and Exp 2 (**B**), pollen grains collected from Lubuski plants grown under four types of water regime in Exp 1 (**C**) and Exp 2 (**D**). Mean values (with standard errors) of pollen morphology-related traits: pollen grain area (**E**), perimeter (**F**), width (**G**), and length (**H**). Letters denote groups of similar mean values obtained by the FPLSD test at *p* < 0.05.

The ANOVA results showed significant effects of treatment and G × T interaction on all studied traits associated with pollen morphology in Exp 1 and significant effects of genotype, treatment, and G × T interaction on these traits in Exp 2. The late-heading genotype showed higher mean pollen grain area values in the C condition compared to CamB in Exp 1. On the other hand, in Exp 2, higher mean values of this trait were recorded in the C condition for the Syrian genotype (Figure 3). No significant differences between genotypes were noticed in the D condition in Exp 1, whereas in Exp 2 higher mean values of this trait were observed in the D condition for CamB compared to Lubuski. No significant differences in mean pollen grain area values were recorded between the C and D conditions for CamB in Exp 1. The D+GA condition negatively affected the pollen grain area of Lubuski in both types of experiments. Similarly, the mean values of pollen grain perimeter, width, and length decreased in this condition for Lubuski in both types of experiments. 

The ANOVA results showed significant effects of treatments on pollen viability in both types of experiments (Supplementary Table S4). In Exp 1, higher mean values of pollen viability (estimated using method 1—Pv1) were recorded in the C and D conditions for Lubuski compared to CamB (Supplementary Figure S4). On the other hand, the Syrian genotype showed higher mean values for this trait in the D condition compared to the C condition. Similar mean values of this trait were observed for the D+GA condition in both studied genotypes. The mean values of Pv1 recorded for CamB subjected to D+GA condition in Exp 2 were also much higher than D in this type of experiment. Pollen viability evaluation by method 2 (Pv2) revealed that in C condition the studied genotypes were characterized by similar levels of pollen viability in Exp 1. For CamB, higher mean values of this trait were recorded in the D condition (Exp 1 and Exp 2) compared to the values observed for the late-heading genotype. For Lubuski, much higher mean Pv2 values were recorded in the D+TR condition in Exp 1.

#### 3.3.2. Pollen Cytological Observations

**Figure 4:**
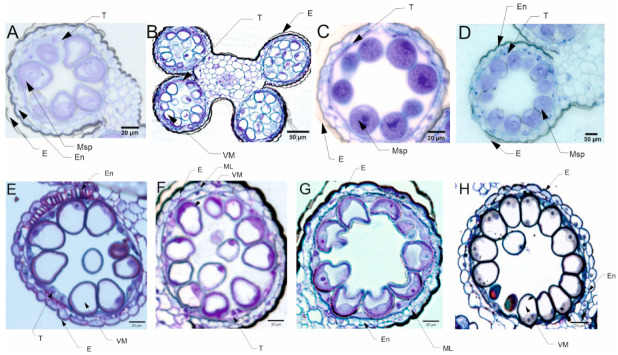
Micrographs showing the pollen grains collected from the late-heading genotype grown under four different water conditions in Exp 1: trinuclear microspores can be seen in the image taken from the tissues of Lubuski plant grown under C condition. In the vegetative cell, the nucleus with well-developed nucleolus and condensed cytoplasm can be observed (**A**). Binuclear, vacuolated microspores can be seen in the image taken from the tissues of Lubuski plant grown under D condition (**B**). Trinuclear microspores are visible in the image taken from the tissues of Lubuski plant grown under D+GA condition. The intensity of toluidine blue staining indicated chromatin condensation in the generative and vegetative nuclei (**C**). Trinuclear microspores can be observed in the image taken from the tissues of Lubuski plant grown under D+TR condition (**D**). Micrographs showing the pollen grains collected from the late-heading genotype grown under four different water conditions in Exp 2: vacuolated microspores with well-developed middle layer can be seen in the image taken from the tissues of Lubuski plant grown under C condition (**E**). Vacuolated microspores can be seen in the image taken from the tissues of Lubuski plant grown under D condition (**F**). Trinuclear microspores are visible in the image taken from the tissues of Lubuski plant grown under D+GA condition (**G**). Binuclear microspores can be ob-served in the image taken from the tissues of Lubuski plant grown under D+TR condition (**H**). VM—vacuolated microspores, E—epidermis, En—endothecium, ML—middle layer, Msp—microspore, and T—tapetum.

**Figure 5:**
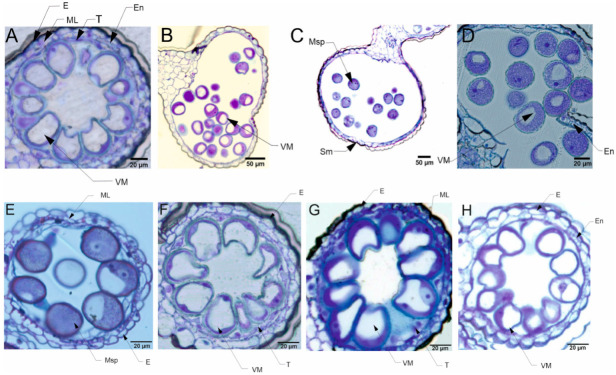
Micrographs showing the pollen grains collected from the early-heading genotype grown under four different water conditions in Exp 1: trinuclear, strongly vacuolated microspores can be seen in the image taken from the tissues of CamB plant grown under C condition (**A**). Trinuclear, partially vacuolated microspores are visible in the image taken from the tissues of CamB plant grown under D condition (**B**). Trinuclear microspores with weak development of vacuoles can be seen in the image taken from the tissues of CamB plant grown under D+GA condition (**C**). Trinuclear microspores are visible in the image taken from the tissues of CamB plant grown under D+TR condition (**D**). Micrographs showing the pollen grains collected from the early-heading genotype grown under four different water conditions in Exp 2: partially vacuolated microspores with well-developed middle layer can be seen in the image taken from the tissues of CamB plant grown under C condition (**E**). Strongly vacuolated microspores are visible in the image taken from the tissues of CamB plant grown under D condition (**F**). Trinuclear microspores are visible in the image taken from the tissues of CamB plant grown under D+GA condition. The intensity of toluidine blue staining indicated chromatin condensation in the generative and vegetative nuclei (**G**). Trinuclear microspores are visible in the image taken from the tissues of CamB plant grown under D+TR condition (**H**). VM—vacuolated microspores, E—epidermis, En—endothecium, ML—middle layer, Msp—microspore, T—tapetum, and Sm—stomium.

The pollen grains collected from plants in the flowering stage (Z65) analyzed by light microscopy (LM) revealed the presence of bi-nucleate or tri-nucleate stages of pollen development and varying degree of cytoplasm vacuolization (Figure 4 and Figure 5).

**Figure 6:**
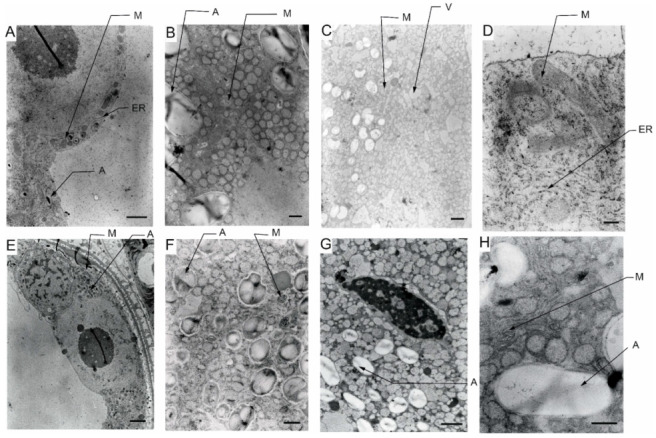
TEM micrographs of pollen grains collected from Lubuski plants grown under control (**A**), drought (**B**), drought+GA (**C**), and drought+TR (**D**) conditions in Exp 1 and under control (**E**), drought (**F**), drought+GA (**G**), and drought+TR (**H**) conditions in Exp 2. ER—endoplasmic reticulum, M—mitochondria with swollen cristae (membrane) structures, A—amyloplast, and V—vacuole. Bars = 2 µm for (**A**,**E**); 1 µm for (**G**); 500 nm for (**B**,**F**,**H**); 200 nm for (**C**,**D**).

Transmission electron microscopy (TEM) observations revealed differences in the cell’s organelle composition and structures between the studied genotypes grown under different conditions. TEM images of pollen grains collected from the Lubuski plant grown under C condition in Exp 1 showed a normal pollen structure; in the cytoplasm, mitochondria with well-developed cristae, amyloplasts with starch grains, and endoplasmic reticulum (ER) (both smooth and rough) were observed (Figure 6). Trinuclear microspores with visible ER structures and numerous other organelles were observed under this condition in Exp 2. The structure of pollen grains collected from the Lubuski plant grown under D condition in Exp 1 showed differentiation in the organization: electron-dense areas with starch-rich amyloplasts and numerous mitochondria with swollen cristae (membrane) structures were localized in the organelle-free cytosol. The ultrastructural observations of the pollen grains collected from Lubuski plants subjected to drought in Exp 2 revealed the presence of starch grains, small vacuoles, and numerous vesicles. Under the D+GA condition in Exp 1, numerous small vacuoles as well as mitochondria with swollen cristae (membrane) structures were observed for this genotype, whereas under this condition in Exp 2 no alterations in the structure of the organelles were noticed. Diluted cytoplasmic areas with few organelles and visible ER structures were observed in pollen grains obtained from Lubuski plants grown under the D+TR condition in Exp 1. Under the same condition in Exp 2, the pollen grains showed no alterations in cell organization but the presence of numerous vesicles and starch grains in the cytoplasm.

**Figure 7:**
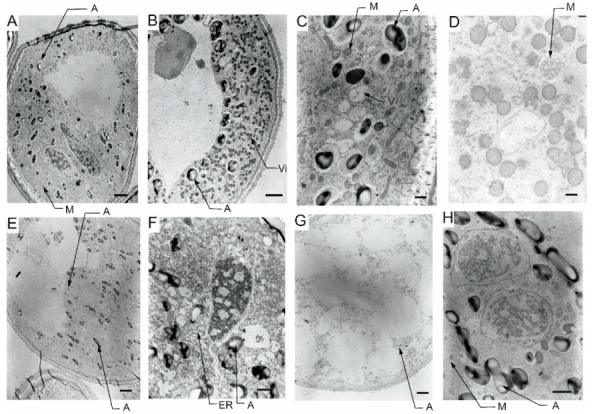
TEM micrographs of pollen grains collected from CamB plants grown under control (**A**), drought (**B**), drought+GA (**C**), and drought+TR (**D**) conditions in Exp 1 and under control (**E**), drought (**F**), drought+GA (**G**), and drought+TR (**H**) conditions in Exp 2. ER—endoplasmic reticulum, M—mitochondria with swollen cristae (membrane) structures, A—amyloplast, Vi—vesicle, and V—vacuole. Bars = 2 µm for (**A**,**B**,**E**,**G**); 1 µm for (**F**,**H**); 500 nm for (**C**); 200 nm for (**D**).

The ultrastructural observations of pollen grains collected from the Syrian genotype grown under optimal (C) water conditions in Exp 1 revealed large vacuoles and mitochondria with well-developed crista and amyloplasts (Figure 7). A similar cell organization, but much more amyloplasts with starch grains, was observed in the pollen grains from the Syrian genotype plants grown in this condition in Exp 2. In D condition in Exp 1, were diluted cytoplasm areas with numerous small vesicles and vacuoles in the pollen grains. In the same water condition in Exp 2, CamB pollen grains showed well-developed ER structures and starch-rich amyloplasts. In the cytoplasm of pollen grains collected from the Syrian genotype grown under D+GA condition in Exp 1, numerous mitochondria and small vacuoles were observed, whereas in the same condition in Exp 2 the pollen grains showed diluted cytoplasm areas with only a few amyloplasts. In the drought condition with the application of the second growth regulator (D+TR), pollen grains collected from CamB plants grown in Exp 1 had diluted cytoplasm areas, mitochondria with swollen cristae (membrane) structures, numerous vesicles and starch grain. Under D+TR condition in Exp 2 trinuclear pollen grains well-developed amyloplasts and mitochondria with swollen cristae (membrane) structures were noticed.

## 4. *HvGAMYB* Transcript Level

**Figure 8:**
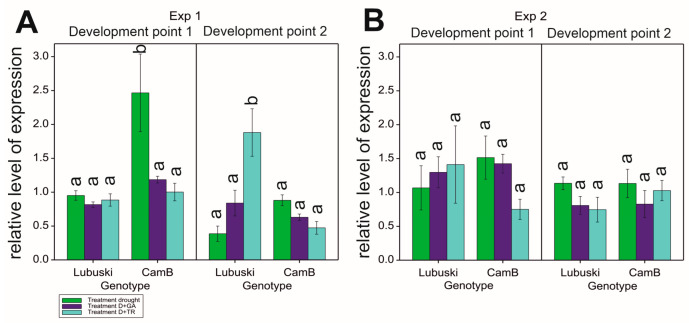
Relative levels of expression (with standard errors) of the *HvGAMYB* gene in two different development points (1—LFE1 and 2—LFE4) in four different water regimes applied in Exp 1 (**A**) and Exp 2 (**B**). Letters denote groups of similar mean values obtained by the FPLSD test at *p* < 0.05.

The results of ANOVA showed significant G × T effects on *HvGAMYB* expression level in the second point of measurement in Exp 1. No significant effects of the variance source on the relative expression level of the studied gene were recorded in Exp 2. In Exp 1, differences in the relative level of expression between the genotypes were noticed in the D condition—for CamB, a much higher relative level of *HvGAMYB* expression was observed in LFE1. During the second developmental point, decreases in the expression of the studied transcription factor was recorded for both genotypes almost in all applied conditions (except for a rapid increase in gene expression recorded for the late-heading genotype in the D+TR condition and a stable level of expression recorded for Lubuski in the D+GA condition) (Figure 8).

## 5. Discussion

Of all the stages in the reproduction process, anther and pollen development are the most sensitive to drought [53]. It has been shown that severe drought, which is already fatal for male development, can only limit female organ development, indicating that these organs are insensitive to drought [54]. Therefore, it is crucial to understand the mechanisms and processes associated with drought-related male sterility to ensure food security. The present study examined morphological and cytoembryological changes in the anther and pollen developmental stages in two spring barley genotypes that differ in earliness and drought tolerance. To the best of our knowledge, this study is the first to explore the ultrastructural pollen alterations in spring barley plants exhibiting contrasting growth habits under drought conditions. 

### 5.1. Differences between Studied Genotypes

Plants with different growth habits adapt to stress environments in different ways [55] and show significant differences in their responses to water scarcity [56]. In particular, barley landraces from regions with challenging climatic conditions near the origin of crop domestication are characterized by valuable drought tolerance traits [57]. In this regard, flowering time is one of the most important agronomic traits that affect both grain yield and quality. The Syrian breeding line CamB, studied here, exhibited extraordinary development by achieving reproductive success as early as possible. Its early-heading plants showed a flag leaf phase even before the onset of tillering. This phenomenon was also noticed in our previous studies on the genotypes of Syrian origin [36,37,58,59]. Lubuski is an old cultivar adapted to East European conditions, where water availability in the past was different than it is now. In this environment, plants follow a stable growth pattern: during the tillering process, biomass develops, the number of stems increases, and then stem elongation begins. After the simultaneous development of many stems, the flowering process is initiated. CamB plants adapted to arid conditions show a different growth strategy: tillering is postponed, and plants use all available resources (water and nutrition) to promote the rapid development of the main stem with the spike. After the initiation of flowering and the development of seeds, which guarantee reproductive success, the early-heading genotype starts to develop more tillers. The adjusted developmental pattern may be beneficial under stressful environments where water conditions are unfavorable almost every day [60]—in this case, the plants have a short life cycle which allows their successful adaptation to different abiotic stresses. As frequent episodes of drought driven by climate change are a real threat to agriculture and food security, DE seems to be a promising strategy that can be adopted by plants, which enables them to improve crop yield and performance under unfavorable water conditions.

The studied genotypes also varied in spike morphology as this trait is strictly linked to the plant growth pattern [61]. Interestingly, for CamB, similar mean values of NGSm were recorded under C and D conditions (Exp 1), but the mean WGSm values were lower in D, which suggests that this genotype did not reduce the number of seeds under stress conditions but the quality of seeds was probably negatively affected by unfavorable conditions. These findings highlight the ability of arid-tolerant plants to adapt to the water condition, which is inconsistent with the general fact that drought limits the number of seeds in barley [62]. However, considering early heading as a drought resistance strategy, it is important to consider the yield quality from an economic point of view.

Chlorophyll fluorescence has long been used in many studies as a practical and sensitive indicator of stress responses in various plant species (e.g., [63,64,65]), including barley [37,66]. In the present study, the ANOVA results showed significant effects of genotype and development points on chlorophyll fluorescence parameters associated with specific energy fluxes per reaction center (RC) (ABS_RC, TRo_RC, Dlo_RC in Exp 1 and ABS_RC, TRo_RC, ETo_RC in Exp 2), which suggests the differences in the drought response strategy of the studied plants and also highlights the negative impact of prolonged stress on plant performance. As reported in [67], drought reduces the number of active RCs, while further steps of photosynthetic electron transfer are less affected, which could explain the findings of the present study. In addition, changes in, for example, ABS_RC recorded here may suggest that the early-heading genotype responds differently to the initial phase of drought stress. However—thereafter—alterations in RCs also occur in this plant. Our study confirmed that the chlorophyll fluorescence induction (OJIP) parameters are very sensitive to drought effects compared to changes in RWC in leaves. These parameters reflect the balance between water supply to the leaf tissue and transpiration rate, and thus are considered as an important indicator of plants’ water status [68]. The results of chlorophyll fluorescence analysis followed by OJIP testing corresponded well with the RWC measurements. Although the mean values of this trait recorded in our study decreased in stress conditions, no significant differences were noticed between the stress treatments for both genotypes, which contrasts with the OJIP test showing significant effects of genotype, treatment, and G × T interaction for some parameters. 

All these findings suggest that the genotypes studied here are characterized by different growth patterns and drought response strategies (in terms of both yield and chlorophyll fluorescence kinetics), thus constituting a promising model to explore the differences in pollen development under drought conditions.

### 5.2. Spike Fertility and Anther Morphology

In the present study, significant effects of treatment were observed for the traits associated with spike fertility (FSm and FSl), only in Exp 1. In the experiment with temporary light deprivation, no significant effects of different water conditions were recorded, which suggests that the dark stress affected traits associated with spike fertility and contributed to the limitation of drought effects on spike morphology. Like several previous studies demonstrating the negative impact of light distortion on the vegetative [69,70] and generative cycle of plant development and other different aspects of plant biology [71,72], the present study also revealed that temporary light deficiency is a stronger stressor for seed development than drought occurrence. Interestingly, the drought condition—applied in both Exp 1 and Exp 2—contributed positively to FSm with increased values recorded for CamB. This can be explained by the fact that this early-heading plant adapted an extraordinary survival strategy that guarantees its wide adaptation in many morphological, biochemical, and physiological aspects to unfavorable conditions. In Exp 2, relatively higher mean FSl values were recorded in C, D, and D+GA conditions for CamB, which can be related to the development of secondary tillers and highlight the effective adaptation of CamB to drought. This is consistent with the tiller development pattern, a highly adaptive process that depends on environmental factors that can promote or suppress lateral shoot development through a complex network of hormonal and regulatory signals [73].

### 5.3. Pollen Viability and Micromorphology

Pollen viability highly depends on the ability of plants to accumulate enough reserve metabolites, especially carbohydrates [74]. Due to drought-induced disruption in the enzymatic processes associated with starch synthesis [75], pollen viability decreases rapidly in plants subjected to water scarcity. Our study only partially confirmed this phenomenon because no differences between C and D conditions were observed for Lubuski—evaluation of pollen viability using method 1 showed decreases in Pv1 only when additional plant growth regulators were applied. This suggests that starch accumulation in Lubuski seeds was not negatively affected by drought but when there was some disruption in plant development, a rapid decline in pollen viability was noticed. Moreover, better pollen viability (Pv1) recorded for CamB in the D condition than in the C condition also showed the adaptation of this genotype to unfavorable water conditions as pollen grains are extremely vulnerable to moisture losses [76]. TTC reacts with dehydrogenase in pollen of plants growing in optimal conditions and appears red, which is considered the most common [77] and indicates enzyme activity in the pollen grains collected from plants grown under different abiotic stresses. Evaluation of pollen viability using method 2 showed the negative impact of all types of drought conditions on Pv2 but still relatively high mean values of this trait were recorded for CamB, suggesting that the Syrian genotype maintained relatively high pollen viability in drought.

The effects of genotype on pollen grain micromorphology were obtained only in Exp 2, which demonstrated the impact of light deprivation on the reproductive phase. Consequently, the pollen morphology variations of plants varied in terms of earliness. Moreover, CamB pollen grain size and shape were stable even under a combination of drought and application of an artificial growth regulator, which is contrary to our expectations as any perturbations in plant development subjected to drought may additionally damage the cell structures. For Lubuski, almost all traits associated with pollen grain morphology were negatively affected by GA application in drought conditions which clearly shows that the pollen grain of the late-heading genotype was vulnerable to the negative impact of growth acceleration. The pollen alterations also had an impact on spike fertility, as in this type of condition, a rapid decrease in mean FSm values was noticed for the late-heading genotype in Exp 1.

### 5.4. Pollen Cytological Observations—LM

For all four types of treatments, trinuclear microspores were observed in pollen grains, which indicates that the development of the Syrian genotype’s pollen was not negatively affected under drought conditions, or this genotype could activate some repair processes. Interestingly, strongly vacuolated microspores were noticed in pollen from plants grown in C and D conditions. It has been shown that vacuolization always occurs during pollen development [78], which suggests that the Syrian genotype is adapted to arid conditions to the extent that under drought conditions the cell structure of pollen grains was almost similar to the pollen collected from plants grown under well-watered condition. Contrary to the observations for CamB, for Lubuski extremely vacuolated, binuclear microspores were observed in pollen for D condition. In a partially dehydrated state, pollen exhibits reduced metabolic activity, lives longer and can better tolerate further desiccation during dispersal [76,79]. Pollen dehydration imposes developmental arrest on maturing pollen, and shifts in the timing of developmental arrest might prevent or enable pollen cell cycle progression to the tricellular condition before dispersal [80]. Altogether this suggests that a delay occurred in pollen development in the Lubuski plant as in LFE4 trinuclear pollen should be present [35] or some perturbations have occurred in the cell division process causing rapid dehydration of the mother plant in drought conditions. The vacuolization process observed in our study was expected as plants under abiotic stress can modulate their development and growth by altering morphological and cellular mechanisms, and cells’ responses to stress might involve changes in the distribution and sorting of specific proteins and molecules. Hence, as vacuoles serve physical and metabolic functions and are essential for cellular responses to general cell homeostasis [81], as well as to abiotic and biotic stresses [82], changes in the number or structure of these organelles are expected in drought conditions. It seems that the artificial regulation of development (GA) did not affect the reaction of CamB to drought, whereas in this condition (D+GA, Exp 1) pollen grains from Lubuski plants showed chromatin condensation, which may suggest the intensification of cellular processes associated with metabolism remodeling as chromatin rearrangements occur in plant cells subjected to drought [83,84]. Interestingly, in Exp 2 pollen development in samples collected from Lubuski plants did not seem to be affected by any applied stress conditions, whereas in samples obtained from CamB plants grown under the same stress conditions processes associated with chromatin condensation can be visible. 

### 5.5. Pollen Ultrastructure Observations—TEM

The effects of the applied treatment on the pollen cellular architecture of the two studied genotypes were studied in detail by TEM. The observations indicated that under adverse conditions, the destruction of organelle structures eventually led to a decrease or even loss of cell physiological function [85]. Similar to previous studies reporting the presence of several sterile pollen grains with diluted cytoplasm and reduced starch under water-deficient conditions [54], in our study (Exp 1) pollen grains from both the studied genotypes showed diluted cytoplasm with an accumulation of numerous, small vesicles. The functions of these vesicles under drought conditions remain elusive but the appearance of these organelles could be affected by metabolism remodeling triggered by stress as the transport of cargo molecules between compartments is mainly carried out by vesicle shuttles (i.e., transport vesicles) [86]. The findings for previously analyzed parameters indicated that CamB reacted similarly to both C and D conditions, but ultrastructural observations showed that changes associated with drought-induced reactions can be triggered at the cellular level. Interestingly, as suggested by TEM micrograph observations, pollen grains obtained from CamB plants subjected to D+GA conditions did not seem to be affected by drought. This phenomenon was probably linked to the acceleration of plant development resulting in a faster onset of second developmental stages (LFE4) in plants grown under this condition compared to plants grown under D conditions. Consequently, D+GA plants were exposed only for a shorter time to stress conditions compared to CamB plants grown under D conditions. On the other hand, under D conditions with the application of the second growth regulator (TR), CamB was subjected for a prolonged time to stress (but the pollen development process was completed) and the TEM observations revealed numerous signs of cellular damage: diluted cytoplasm, accumulation of vesicles and presence of mitochondria with swollen cristae (membrane) structures. The study conducted on wheat [87] revealed that under drought conditions mitochondria were swollen and vacuolized, which can be observed on TEM micrographs of pollen grains collected from CamB plants subjected to D+TR condition. Some studies suggest that developmental alterations can be irreversible [88,89] and highlight the negative effects of growth modification applied under drought in plants adapted to arid conditions. Although the extent of mitochondrial damage in plants grown under drought is hard to specify [90], it is probably related to the species and variety of plants [91]. These types of mitochondrial alterations were also observed in pollen grains from Lubuski plants grown under D and D+GA conditions in Exp 1 in our study. Interestingly, no swollen mitochondrial membranes were noticed on TEM micrographs for CamB plants grown under D conditions in this type of experiment. The cellular organization was not that much affected by drought as the organelles of pollen grains collected from Lubuski plants subjected to drought in Exp 1. Diluted cytoplasm structures were observed in the micrographs of pollen grains taken from CamB plants grown under D+GA condition in Exp 2, contrary to Exp 1, where similar cellular alterations were noticed for D+TR conditions. This was probably associated with the changes in plant development triggered by growth regulators—in Exp 2 acceleration in plant development caused the CamB plants to be subjected to light deprivation conditions from the very beginning stress duration, whereas the plants grown under C, D, and D+TR conditions modified their growth which allowed the completion of pollen grain development when dark stress was over. Although, the presence of dense systems of ER on the TEM micrographs of CamB plants grown under D conditions in Exp 2 may suggest the same perturbations in pollen development as abiotic stress can lead to misfolding of proteins and their accumulation, causing an ER stress situation [92,93]. This may be linked to the modification of plant development by light deprivation stress. In Exp 1 for the late-heading genotype, drought-induced alterations were observed in organelle structures—mitochondria with swollen membrane structures in D and D+GA conditions and diluted cytoplasm areas with a small number of cell organelles. The presence of dense systems of ER was noticed on the micrographs of Lubuski plants grown under D+TR condition in Exp 1, which showed that some processes related to cellular metabolism modification were triggered. This can be explained by prolonged exposure of plants to drought but did not provide enough information about the disruption of the pollen development process. Surprisingly, no such cellular changes were observed on the TEM micrographs of Lubuski plants grown under the same condition in Exp 2. This was presumably associated with the late growth habit of Lubuski and the delay of the development process, especially under drought—the plants limited the speed of development and extended the period between light deprivation and the flowering process. Moreover, stress exposure of mother plants did not affect the development of spikes in the secondary tillering process, which indicated that Lubuski plants managed to obtain enough nutrition to complete pollen development after drought. 

### 5.6. HvGAMYB Expression Fluctuation

The present study confirmed the expression of *HvGAMYB* in the anther tissues of plants subjected to different water conditions. Results from the study published previously [94] showed that a decrease in anther size was linked to the increase in GAMYB levels, particularly a decrease in anther length. These findings are in line with the results of the present study, in which CamB plants with a higher level of *HvGAMYB* expression and lower mean values of anther width were recorded compared to Lubuski plants under the same stress treatment. In our study, *HvGAMYB* was expressed at a relatively high level in anthers collected from the CamB plants subjected to drought in LFE1. Transgenic barley plants with a much higher level of GAMYB protein in their anthers were shown to be male sterile [94,95]. In our study, the relatively high expression level of this transcription factor in anthers collected from CamB plants cannot be linked to pollen—even partially—sterility as in D condition the traits associated with pollen morphology and pollen viability did not seem to be affected by drought. Noteworthy, under D conditions with the application of a growth regulator, the relative expression levels of *HvGAMYB* were lower compared to those recorded under drought conditions without spraying interactions, which emphasized the complex nature of the expression fluctuations of some transcription factors in terms of plant growth modifications under drought condition. Surprisingly, *HvGAMYB* expression was relatively high in tissues collected from Lubuski plants grown under D+TR in LFE4. This condition should have resulted in plant development delay—this phenomenon can be linked to the involvement of this gene in pollen and anther development process [94] in spike tissues developing during secondary tillering. The results of this study clearly showed that plant development perturbations affected the circadian rhythm functions and the expression of transcription factors, which can be directly linked to the development of generative organs and pollen. Light deprivation applied in Exp 2 did not result in significant differences in *HvGAMYB* expression—contrary to the treatment applied in Exp 1. As *HvGAMYB* is part of the GA-dependent flowering pathway [14], this finding confirmed the existence of a close association between gibberellin and photoperiod pathways [19]. 

## 6. Conclusions

This study clearly showed that the duration of the vegetation period has an influence on plants’ responses to drought conditions as every modification can change the dynamics of drought effects as well as the duration of a plant’s exposure to drought. As a consequence, plants exhibited different drought reactions which also had an impact on pollen development. When the mother plant experiences stress conditions, the signal will reach pollen grains and affect pollen preparation and the timing of pollen dispersal [96,97]. The results of our study suggest that this conclusion is partially correct—in some cases, the exposure of the mother plant to drought did not seem to severely affect pollen grain development in spikes that elude (by growth delay or secondary tillering process) the unfavorable conditions and their consequences, even in plants not adapted to arid condition. In our study, CamB and Lubuski mother plants were affected by drought which was confirmed by the evaluation of yield performance and chlorophyll fluorescence kinetics. However, the pollen development of plants grown under specific conditions (e.g., Lubuski in Exp 2) was not much affected, contrary to our expectations. Even though no damage in cellular components was noticed in pollen grains for the late-heading genotype in TEM observations, the evaluation of pollen viability indicated low pollen quality after dispersal. An important indicator of proper pollen development may also be the two proposed traits associated with spike fertility, which showed clearly a negative impact of drought on the generative process, and consequently, seed development. According to phenology observations for Exp 2, the timing between the flag leaf stage (where stresses begin) and flowering was extremely short for CamB plants, due to which this genotype completed the flowering process under light deprivation treatment. At the same time, the late-heading genotype delayed generative development and, probably, recovered to some extent, which can be observed in TEM and LM micrographs. 

This study revealed the ultrastructural changes in anther and pollen grains of two contrasting barley genotypes—well characterized by our previous investigations—and showed that alteration in cellular organizations can be related to the duration of stress exposition. The results of this study also suggest that growth modification, as well as perturbations in light distribution, can affect the *HvGAMYB* expression. As these transcription factors should be expressed in a tailored pattern, the changes in the expression can have a significant impact on many development-linked processes. 

## Figures and Tables

**Table 1:** List of phenotypic traits with description, their abbreviations (abbrev.) and measurement techniques.

Trait (Unit), (Abbrev.)	Trait Description
Total number of tillers, (Tn)	Number of tillers with fertile and non-fertile (without grains) spikes
Number of productive tillers, (PTn)	Number of tillers with fertile spikes
Length of main spike (cm), (LSm)	Length of main spike from 10 randomly selected spikes in a pot (without awns)
Number of spikelets per main spike, (NSSm)	Number of spikelets in spike of main stem-average e for 10 main spikes in a pot
Number of grains per main spike, (NGSm)	Number of grains collected from one spike of main stem—average for 10 main spikes in a pot
Weight of grains per main spike, (WGSm)	Weight of grain collected from one spike of the main stem—average for 10 main spikes in a pot
Length of lateral spike (cm), (LSl)	Length of spike from lateral stem—average for 10 lateral spikes in a pot (without awns)
Number of spikelets per lateral spike, (NSSl)	Number of spikelets per spike of lateral stem—average for 10 lateral spikes in a pot
Number of grains per lateral spike, (NGSl)	Number of grains collected from spike of lateral stem—average for 10 lateral spikes in a pot
Weight of grains per main spike, (WGSl)	Weight of grain collected from one spike of the lateral stem—average for 10 lateral spikes in a pot
Grain yield (g), (GY)	Average weight of grains collected from one plant, calculated as average of measurements of grain weight for 10 plants
Thousand grain weight (g), (TGW)	Average weight of 1000 grains, calculated as average of 1000 × average weight of one grain for 20 spikes in a pot
Fertility of the main spike, (FSm)	NGSm/NSSm ratio
Fertility of the lateral spike, (FSl)	NGSl/NSSl ratio

**Table 2:** Chlorophyll fluorescence induction (OJIP) parameters calculated in the study (with abbreviations).

Trait	Abbrev.
Quantify the PSII behavior were the absorbed energy flux	ABS_RC
Trapped energy flux	TRo_RC
Electron transport flux	Eto_RC
Dissipated energy flux	DIo_RC
Maximum quantum yield of primary photochemistry	Fv_Fm
Probability/efficiency that a trapped exciton moves an electron into the electron transport chain beyond QA	Ψ_o
Quantum yield of electron transport	Φ_Eo
Probability that the energy of an absorbed photon is dissipated as heat	Φ_Do
Performance index	Pi _Abs

## Data Availability

Not applicable.

## Supplementary Materials

The following supporting information can be downloaded at: https://www.mdpi.com/article/10.3390/cells12121656/s1, Table S1: Crop growth and development stages according to the Zadoks scale; Table S2: The dates of sample collection for microscopic observation conducted in both experiments (Exp 1 and Exp 2. Treatments: C—control condition; D—drought condition; D+GA—drought condition combined with GA3 application; D+TR—drought condition combined with Trinexapac application. NoD—number of days, the first day is the day of droughts beginning; Table S3: DNA sequences of the gene specific primers; Table S4: Results of analysis of variance for observed traits (*p* values for testing significance of variation sources); Figure S1: Mean values of yield-related traits (with standard errors); Figure S2: Mean values (with standard errors) recorded for chlorophyll fluorescence parameters in both type of experiments at two development points (LFE1 and LFE3); Figure S3: Means values (with standard errors) of RWC recorded for two contrasting genotypes in different water regimes in both type of experiments; Figure S4: Mean values (with standard errors) of pollen viability evaluation traits (two different methods: A—KI/I2—Pv1; B—TTC—Pv2) recorded for two contrasting genotypes in different water regimes in both type of experiments.
