# Supplementary material for: The Effects of Growth Modification on Pollen Development in Spring Barley (Hordeum vulgare L.) Genotypes with Contrasting Drought Tolerance
Source: Cells. 2023 Jun 18;12(12):1656. doi: 10.3390/cells12121656 (PMC10297496; doi:10.3390/cells12121656)
Supplement: Supplementary file 1 [file cells-12-01656-s001.zip › Supplementary Figure S2.pdf]

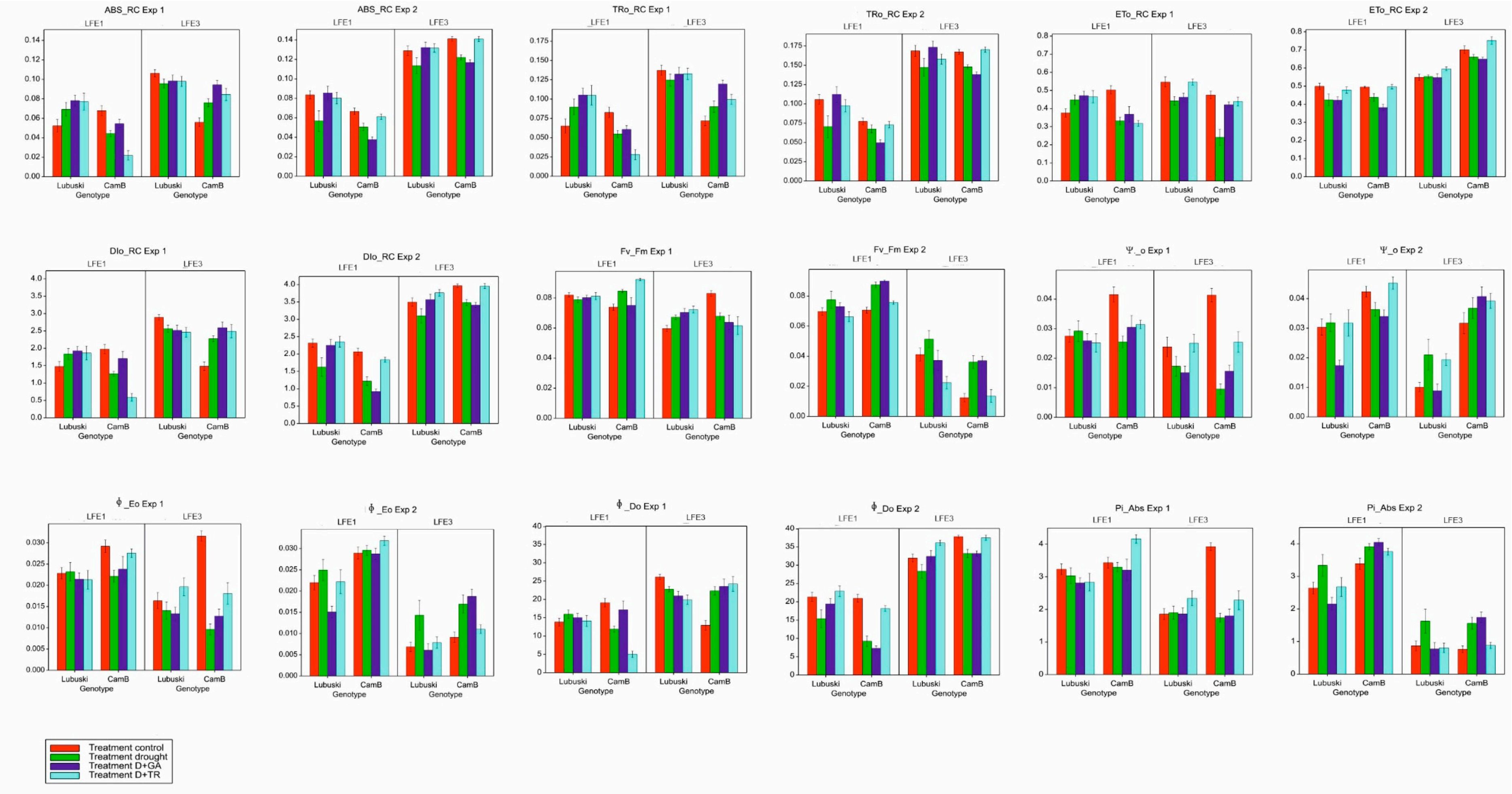

Supplementary Figure S2. Mean values (with standard errors) recorded for chlorophyll fluorescence parameters in both type of experiments at two development points (LFE1 and LFE3).
