# Supplementary material for: The Effects of Growth Modification on Pollen Development in Spring Barley (Hordeum vulgare L.) Genotypes with Contrasting Drought Tolerance
Source: Cells. 2023 Jun 18;12(12):1656. doi: 10.3390/cells12121656 (PMC10297496; doi:10.3390/cells12121656)
Supplement: Supplementary file 1 [file cells-12-01656-s001.zip › Supplementary Figure S4.pdf]

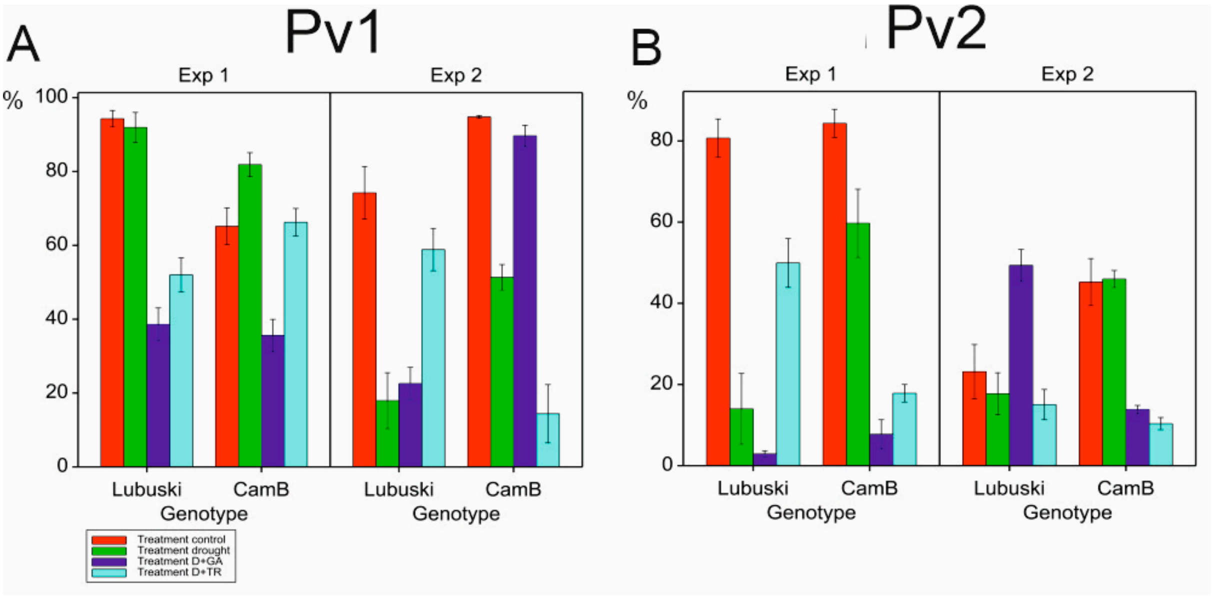

Supplementary Figure S4. Mean values (with standard errors) of pollen viability evaluation traits (two different methods: A – KI/I2 – Pv1; B – TTC - Pv2) recorded for two contrasting genotypes in different water regimes in both type of experiments.
