# Supplementary material for: The Effects of Growth Modification on Pollen Development in Spring Barley (Hordeum vulgare L.) Genotypes with Contrasting Drought Tolerance
Source: Cells. 2023 Jun 18;12(12):1656. doi: 10.3390/cells12121656 (PMC10297496; doi:10.3390/cells12121656)
Supplement: Supplementary file 1 [file cells-12-01656-s001.zip › Supplementary Table S1.pdf]

Supplementary Table S1. Crop growth and development stages according to the Zadoks scale.

Primary growth stages	Name of the secondary stages
0 - Germination	00 - Dry seed
	01 - Start of water absorption
	03 - Seed fully swollen
	05 - First root emerged from seed
	07 - Coleoptile emerged from seed
	09 - First green leaf just at tip of coleoptile
1 - Seedling growth	0 - First leaf through coleoptile
	11 - First leaf emerged
	12 - Two leaves emerged
	13 - Three leaves emerged
	14 - Four leaves emerged
	15 - Five leaves emerged
	16 - Six leaves emerbed
	17 - Seven leaves emerged
	18 - Eight leaves emerged
	19 - Nine or more leaves emerged
2 - Tillering	20 - Main stem only
	21 - Main stem and one tiller
	22 - Main stem and two tillers
	23 - Main stem and three tillers
	24 - Main stem and four tillers
	25 - Main stem and five tillers
	26 - Main stem and six tillers
	27 - Main stem and seven tillers
	28 - Main stem and eight tillers
	29 - Main stem and nine or more tillers
3 - Stem elongation	30 - Pseudostem (youngest leaf sheath erection)
	31 - First node detectable
	32 - Second node detectable
	33 - Third node detectable
	34 - Fouth node detectable
	35 - Fifth node detectable
	36 - Sixth node detectable
	37 - Flag leaf just visible
	39 - Flag leaf ligule just visible
4 - Booting	41 - Flag leaf sheath extending
	43 - Boots just visible swollen
	45 - Boots swollen
	47 - Flag leaf sheath opening
	49 - First awns visible
5 - Ear emergence from boot	51 - Tip of ear just visible
	53 - Ear quarter emerged
	55 - Ear half emerged
	57 - Ear three quarters emerged

	59 - Ear emergence complete
6 - Anthesis	61 - Beginning of anthesis (few anthers at middle of ear)
	65 - Anthesis half-way (anthers occurring half way to tip and base of ear)
	69 - Anthesis complet
7 - Milk development	71 - Kernel water ripe
	73 - Early milk
	75 - Medium milk
	77 - Late milk
8 - Dough development	83 - Early dough
	85 - Soft dough
	87 - Hard dough
9 - Ripening	91 - Grain hard, difficult to divide
	92 - Grain hard, not dented by thumbnail
	93 - Grain loosening in daytime
	94 - Over-ripe straw dead and collapsing
	95 - Seed dormant
	96 - Viable seed giving 50% germination
	97 - Seed not dormant
	98 - Secondary dormancy induced
	99 - Secondary dormancy lost
