# Supplementary material for: The Effects of Growth Modification on Pollen Development in Spring Barley (Hordeum vulgare L.) Genotypes with Contrasting Drought Tolerance
Source: Cells. 2023 Jun 18;12(12):1656. doi: 10.3390/cells12121656 (PMC10297496; doi:10.3390/cells12121656)
Supplement: Supplementary file 1 [file cells-12-01656-s001.zip › Supplementary Table S2.pdf]

Supplementary Table S2. The dates of sample collection for microscopic observation conducted in both experiments (Exp 1 and Exp 2). Treatments: C – control condition; D – drought condition; D+GA - drought condition combined with GA3 application; D+TR - drought condition combined with Trinexapac application. NoD – number of days, the first day is the day of droughts beginning. The number of days NoD_1, NoD_2 and NoD_3 refer to the appearance of the LFE1, LFE3 and LFE4 stages, respectively, starting from flag leaf stage (drought start).

Genotype	Treatment	Exp 1			Exp 2		
		NoD_1	NoD_2	NoD_3	NoD_1	NoD_2	NoD_3
CamB	C	2	5	7	6	11	16
	D	3	6	9	7	13	17
	D+GA	2	4	5	3	7	8
	D+TR	4	8	11	7	14	20
Lubuski	C	5	10	12	8	20	24
	D	6	11	14	9	20	25
	D+GA	5	8	12	8	15	19
	D+TR	7	12	17	9	22	27
