# Supplementary material for: The Effects of Growth Modification on Pollen Development in Spring Barley (Hordeum vulgare L.) Genotypes with Contrasting Drought Tolerance
Source: Cells. 2023 Jun 18;12(12):1656. doi: 10.3390/cells12121656 (PMC10297496; doi:10.3390/cells12121656)
Supplement: Supplementary file 1 [file cells-12-01656-s001.zip › Supplementary Table S3.pdf]

Supplementary Table S3. DNA sequences of the gene specific primers.

Gene	Sequence (5′→3′)	Ampl. length (bp)	Melt temp. [ºC]	E [%]
HvGAMYB	F: CCTTCACTCCAAGATACCGAAT	89	63	108.3
	R: GGATCAACCAACTCCGTAGG		63	
ACT1	F: GCCGTGCTTTCCCTCTATG	235	63	101.8
	R: GCTTCTCCTTGATGTCCCTTA		63	
UBI	F: TCGCCGTCCTCCAGTTCTAC	63	63	105.1
	R: CCTTCCTGAGCCTGGTTACCT		63	
UPL	F: CTGAAGAGTTAGGCGGGAAA	100	63	105.8
	R: ATCGCATGAACGTAGTGCAA		63	
