# Supplementary material for: The Effects of Growth Modification on Pollen Development in Spring Barley (Hordeum vulgare L.) Genotypes with Contrasting Drought Tolerance
Source: Cells. 2023 Jun 18;12(12):1656. doi: 10.3390/cells12121656 (PMC10297496; doi:10.3390/cells12121656)
Supplement: Supplementary file 1 [file cells-12-01656-s001.zip › Supplementary Table S4.pdf]

Supplementary Table S4. Results of analysis of variance for observed traits (P values for testing significance of variation sources).

Group of traits	Trait	Experiment 1 (Exp 1)						Experiment 2 (Exp 2)					
		Genotype (G)	Treatment (T)	Development point (DP)	G × T interaction	G × DP	T × DP	Genotype (G)	Treatment (T)	Development point (DP)	G × T interaction	G × DP	T × DP
Phenology	Tillering	0.003	0.500	-	-	-	-	0.003	0.500	-	-	-	-
	Flag leaf	< 0.001	0.500	-	-	-	-	< 0.001	0.500	-	-	-	-
	Flowering	< 0.001	0.500	-	-	-	-	< 0.001	0.500	-	-	-	-
	Heading	0.004	0.082	-	-	-	-	0.014	0.187	-	-	-	-
Phenotype	Tn	0.819	< 0.001	-	0.044	-	-	0.242	< 0.001	-	< 0.001	-	-
	PTn	< 0.001	< 0.001	-	0.001	-	-	0.418	0.001	-	< 0.001	-	-
	LSm	< 0.001	< 0.001	-	< 0.001	-	-	< 0.001	0.001	-	0.007	-	-
	NSSm	< 0.001	0.003	-	< 0.001	-	-	< 0.001	0.002	-	0.038	-	-
	NGSm	< 0.001	< 0.001	-	< 0.001	-	-	< 0.001	0.015	-	< 0.001	-	-
	WGSm	< 0.001	< 0.001	-	< 0.001	-	-	< 0.001	< 0.001	-	< 0.001	-	-
	LSl	< 0.001	< 0.001	-	0.195	-	-	< 0.001	0.002	-	0.184	-	-
	NSSl	< 0.001	0.001	-	0.234	-	-	< 0.001	< 0.001	-	< 0.001	-	-
	NGSl	0.002	< 0.001	-	0.007	-	-	0.480	0.020	-	< 0.001	-	-
	WGSl	0.087	< 0.001	-	< 0.001	-	-	0.049	0.021	-	< 0.001	-	-
	GY	0.514	< 0.001	-	< 0.001	-	-	0.733	0.001	-	< 0.001	-	-
	TGW	0.541	< 0.001	-	0.053	-	-	0.016	< 0.001	-	< 0.001	-	-
	FSm	0.284	< 0.001	-	< 0.001	-	-	0.013	0.115	-	0.015	-	-
	FSl	0.224	< 0.001	-	0.003	-	-	< 0.001	0.041	-	< 0.001	-	-
Chlorophyll fluorescence	ABS_RC	< 0.001	0.051	< 0.001	0.162	0.879	0.208	< 0.001	< 0.001	< 0.001	< 0.001	< 0.001	0.893
	TRo_RC	< 0.001	0.049	< 0.001	0.074	0.816	0.454	< 0.001	< 0.001	< 0.001	< 0.001	0.001	0.974
	ETo_RC	< 0.001	< 0.001	0.004	< 0.001	0.065	0.002	< 0.001	< 0.001	< 0.001	0.113	< 0.001	0.045
	DIo_RC	< 0.001	0.051	< 0.001	0.150	0.986	0.014	0.011	< 0.001	< 0.001	< 0.001	< 0.001	0.455

	Fv_Fm	0.446	0.294	< 0.001	0.034	0.810	0.036	0.295	< 0.001	< 0.001	0.001	< 0.001	0.063
	Ψ_o	0.005	< 0.001	< 0.001	< 0.001	0.335	0.001	< 0.001	< 0.001	< 0.001	0.009	< 0.001	0.006
	ϕ_Eo	0.002	< 0.001	< 0.001	< 0.001	0.502	0.005	< 0.001	0.001	< 0.001	0.001	0.079	0.002
	ϕ_Do	0.026	0.013	< 0.001	0.059	0.997	< 0.001	0.457	< 0.001	< 0.001	< 0.001	< 0.001	0.238
	Pi_Abs	< 0.001	< 0.001	< 0.001	0.003	0.597	0.019	< 0.001	< 0.001	< 0.001	< 0.001	< 0.001	0.260
RWC		0.470	< 0.001	-	0.132	-	-	0.076	< 0.001	-	0.253	-	-
Anther morphology	Length	< 0.001	< 0.001	-	0.1372	-	-	1	< 0.001	-	0.4106	-	-
	Width	1	< 0.001	-	< 0.001	-	-	< 0.001	< 0.001	-	< 0.001	-	-
Pollen grain morphology	Area	0.682	< 0.001	-	< 0.001	-	-	< 0.001	< 0.001	-	< 0.001	-	-
	Perimeter	0.270	< 0.001	-	< 0.001	-	-	< 0.001	< 0.001	-	< 0.001	-	-
	Width	0.930	< 0.001	-	< 0.001	-	-	< 0.001	< 0.001	-	< 0.001	-	-
	Length	0.193	< 0.001	-	< 0.001	-	-	< 0.001	< 0.001	-	< 0.001	-	-
Pollen viability	Method 1 (Pv1)	0.027	< 0.001	-	0.001	-	-	< 0.001	< 0.001	-	< 0.001	-	-
	Method 2 (Pv2)	0.170	< 0.001	-	< 0.001	-	-	0.409	< 0.001	-	< 0.001	-	-
GAMYB expression	LFE1	0.004	0.010	-	0.024	-	-	0.901	0.678	-	0.247	-	-
	LFE3	0.019	0.015	-	<0.001	-	-	0.594	0.171	-	0.665	-	-
