# Supplementary figures and images for: The Effects of Growth Modification on Pollen Development in Spring Barley (Hordeum vulgare L.) Genotypes with Contrasting Drought Tolerance
Source: Cells. 2023 Jun 18;12(12):1656. doi: 10.3390/cells12121656 (PMC10297496; doi:10.3390/cells12121656)

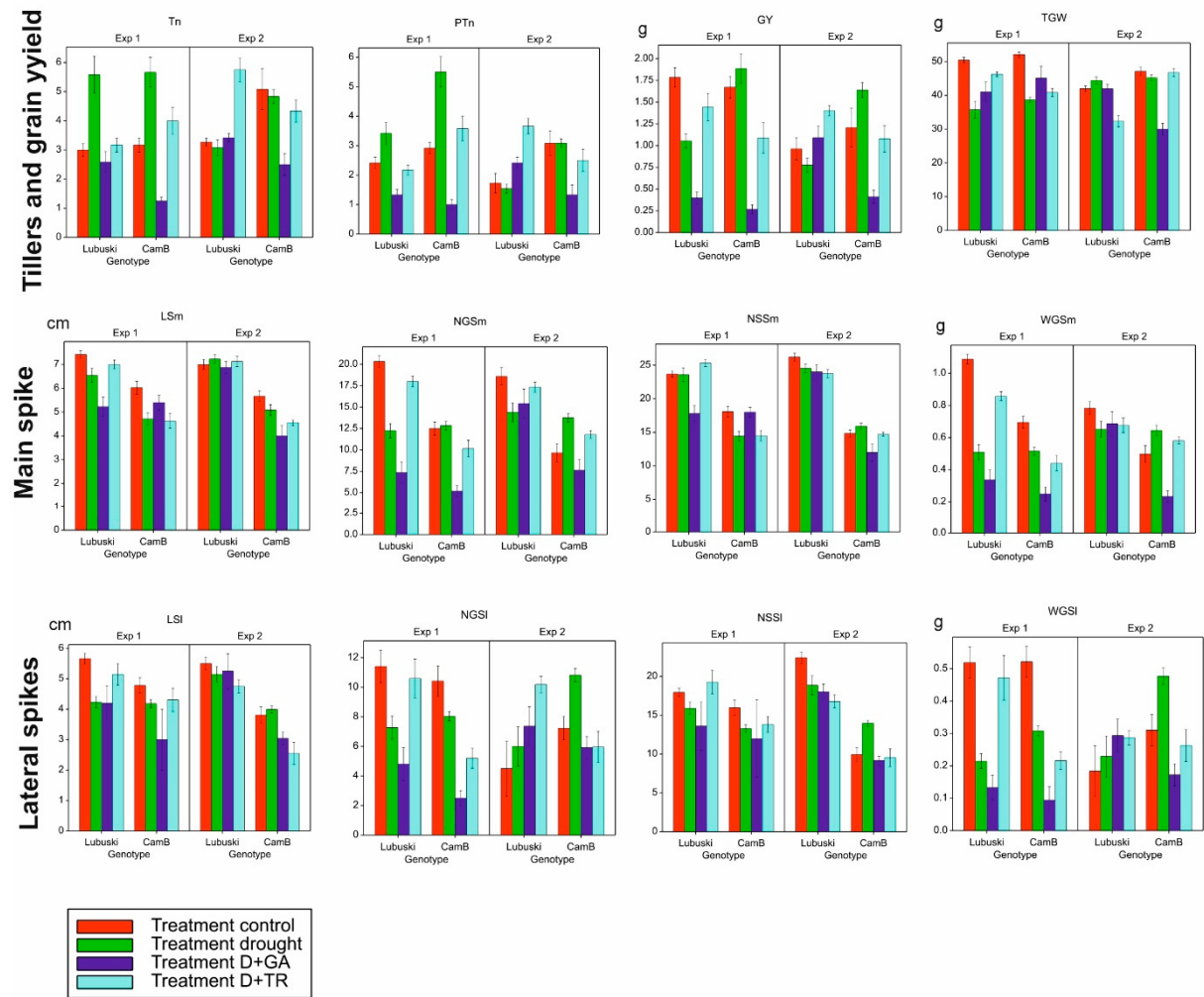

Supplementary Figure S1. Mean values of yield-related traits (with standard errors).

Supplement: Supplementary file 1 [file cells-12-01656-s001.zip › Supplementary Figure S1.pdf]
